# A System Dynamics Model for Ecological Environmental Management in Coal Mining Areas in China

**DOI:** 10.3390/ijerph17062115

**Published:** 2020-03-23

**Authors:** Fulei Shi, Haiqing Cao, Chuansheng Wang, Cuiyou Yao

**Affiliations:** School of Management and Engineering, Capital University of Economics and Business, Beijing 100070, China; shifulei@cueb.edu.cn (F.S.); ycy@cueb.edu.cn (C.Y.)

**Keywords:** ecological environmental management, system dynamics model, coal mining areas

## Abstract

In recent years, mounting attention has been paid to ecological environmental management in coal mining areas in China. This paper conducts a system dynamics (SD) model for ecological environmental management in coal mining areas. Firstly, the whole causal loop diagram of the system is built to illustrate the general system. Secondly, five subsystems are presented according to the causal loop diagram. Then, given the stable investment for ecological environmental management in coal mining areas, our objective is to find a better allocation that can get the best ecological environmental quality in coal mining areas. Notably, we present six allocations of the investment for ecological environmental management in coal mining areas. The results show that, in allocation 4, we can get the best ecological environmental quality in coal mining areas. That is, the best improvement of mining environment can be achieved by distributing the treatment cost highly on the proportion of investment in green vegetation.

## 1. Introduction

In the past few decades, due to the fast economy development and the consequently soaring demand for coal to generate electricity, China has become the largest coal producer and consumer in the world [1]. While coal makes an important contribution to energy generation, its environmental impact has been a challenge [2]. The expansion of the coal mining industry in China has placed high pressure on the ecological environmental management in coal mining areas. As a result, a mounting amount of pollution generated. Such as air pollution, water pollution, solid waste pollution, vegetation destruction, land damage and so on. Therefore, how to effectively improve the quality of the ecological environment in coal mining areas has become an urgent problem to be solved.

In recent years, researches on ecological environmental management in coal mining areas have become a very popular project for scholars to deal with. In one branch, for a review of the literature on policies of ecological environmental management in coal mining areas, see [3,4,5,6,7]. For example, Franks et al. [3] analyzed the cumulative impacts of coal mining on regional communities and environments in Australia. Yu [4] presented a case study in southwest China to learn the role of sustainable coal mining in enhancing environmental quality and combating global environmental change. Mikhailov et al. [5] discussed the ecological risk management in coal mining and processing. Gautam et al. [6] studied particulate matter pollution in opencast coal mining areas. Chabukdhara and Singh [7] made an overview of environmental issues and treatment approaches. Then, he proposed some management and treatment strategies to reduce environmental impacts of coals. However, these literatures only stay at the level of theoretical analysis, lacking effective methods and data support. In another branch, different kinds of methods are used to study the ecological environmental management in coal mining areas, such as modeling and simulation, statistical analysis method, application of GIS, AHP approach and so on, see [8,9,10,11,12,13,14,15,16,17,18]. For example, Kowalska [8] studied the problem of environmental and social risk management during the process of colliery liquidation. Li et al. [9] took Mentougou district of Beijing as a case, discussed the ecosystem service loss of coal mining in China. Based on the application of GIS, Liao et al. [10] evaluated ecological vulnerability in environmental impact assessment of master plan of coal mining area. Mahdevari et al. [11] presented a fuzzy TOPSIS method to study human health and safety risks management in underground coal mines. Shen et al. [13] proposed an AHP approach to explore how the “appropriate implementation approach” and “continuous improvement” are the weaker area in the case of the Indian mining sector. Yu and Gao [17] established a dynamic programming model to discuss environmental investment decision-making in coal mining. Although they used some effective methods to analysis the ecological environmental management in coal mining areas, how to allocate environmental investment in coal mining areas into many ways can get the best effect in the environmental quality has been rarely discussed.

Forrester [19] established system dynamics (SD) model to study the behaviors of feedback loops which contain stocks (levels) and flows. SD provides an effective way for modeling, simulating and studying complex systems. It has been applied to many areas, such as environment, supply chains, water management, transportation and so on [20,21,22,23,24,25,26,27,28,29]. For example, Song et al. [21] proposed a system dynamics model to simulate the land green supply chain, they divided it into four subsystems to simulate the develop trend of technology, energy, environment, and economy in Shandong Province from 2003 to 2020. Chen and Wei [22] discussed the SD model in the application of water security. Golroudbary and Zahraee [23] established a SD model to evaluate the system behavior of an electrical manufacturing company. Sahin et al. [26] presented a SD model that augments the usual water utility representation of the physical linkages of water grids, by adding inter-connected feedback loops in tariff structures, demand levels and financing capacity. Thompson and Bank [28] proposed a SD model to build design and operation, and demonstrates the use of the method for the analysis of a building subjected to a bioterrorist attack. Notably, Yu and Wei [30] presented a hybrid model based on a genetic algorithm and SD for coal production environmental pollution load in China.

To the best of our knowledge, there is little research discussing the topic of ecological environmental management in coal mining areas in China in a SD model. Moreover, the government will make environmental investment in coal mining areas in China, and how to allocate it into many ways can get the best effect in the environmental quality is a complex project to deal with. SD can provide an effective way on this problem. Therefore, in this paper, our contributions can be summarized as follows: (1) Given the stable investment, we propose a SD model for ecological environmental management in coal mining areas to find a better allocation that can get the best ecological environment quality in coal mining areas. (2) We discuss six cases in our study, then, some simulations are given to illustrate the results.

The rest of this paper is organized as follows: In Section 2, we describe in detail about the SD model. In Section 3, we conduct a case study in China. Then, some simulations are proposed. In Section 4, we present the results of our study. In Section 5, we present the discussion to illustrate some contributions and some limitations of the study. In the last Section 6, we discuss the conclusions of the study and introduced some work in the further research.

## 2. The SD Model

In this section, the SD model for ecological environmental management in coal mining areas in China is established.

### 2.1. Causal Relationship of Ecological Environment in Coal Mining Area

**Figure 1:**
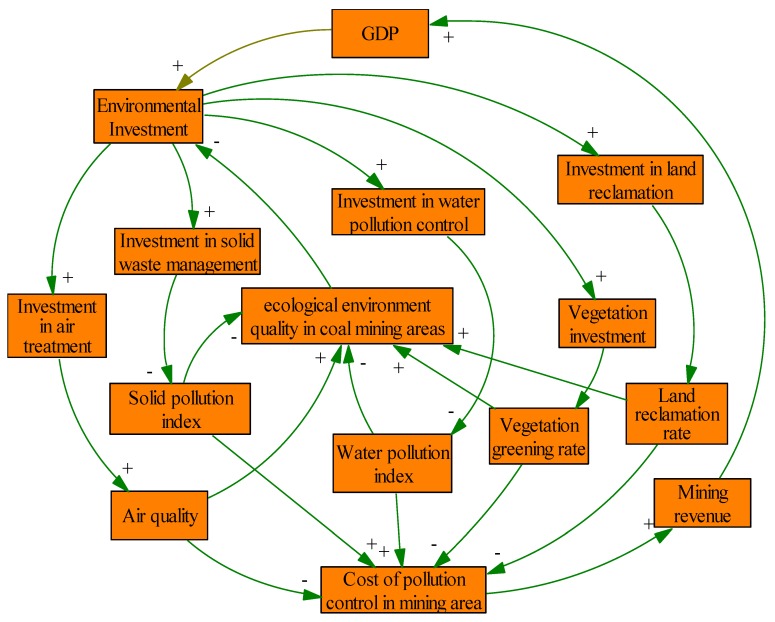
The whole causal loop diagram of the system.

In this paper, we regard the coal mining areas as a whole system. As we all know, there are many subsystems in coal mining areas. To simplify the analysis, we just suppose that there are five subsystems, namely: Atmospheric subsystem, solid waste subsystem, water resource subsystem, vegetation cover subsystem and land reclamation subsystem according to the introduction of problems in coal mining areas [2]. Then, the whole causal loop diagram of the system is shown in Figure 1.

From Figure 1, several causal feedback loops see as follows:

(1) GDP →+ Environmental Investment →+ Investment in air treatment →+ Air quality →– Cost of pollution control in mining area →+ Mining revenue →+ GDP.
(2) GDP →+ Environmental Investment →+ Investment in solid waste management →– Solid pollution index →+ Cost of pollution control in mining area →– Mining revenue →+ GDP.
(3) GDP →+ Environmental Investment →+ Investment in water pollution control →– Water pollution index →+ Cost of pollution control in mining area →– Mining revenue →+ GDP.
(4) GDP →+ Environmental Investment →+ Vegetation investment →– Vegetation greening rate →+ Cost of pollution control in mining area →– Mining revenue →+ GDP.
(5) GDP →+ Environmental Investment →+ Investment in land reclamation →– Land reclamation rate →+ Cost of pollution control in mining area →– Mining revenue →+ GDP.
(6) Environmental Investment →+ Investment in air treatment →+ Air quality →+ ecological environment quality in coal mining areas →– Environmental Investment.
(7) Environmental Investment →+ Investment in solid waste management →+ Solid pollution index →– ecological environment quality in coal mining areas →– Environmental Investment.
(8) Environmental Investment →+ Investment in water pollution control →– Water pollution index →– ecological environment quality in coal mining areas →– Environmental Investment.
(9) Environmental Investment →+ Vegetation investment →– Vegetation greening rate →– ecological environment quality in coal mining areas →– Environmental Investment.
(10) Environmental Investment →+ Investment in land reclamation →– Land reclamation rate →– ecological environment quality in coal mining areas →– Environmental Investment.

From the above loops, we just take loops (1) and (6) as two examples to make an explanation. Loops (1) is a positive feedback loop. A positive feedback loop means that an increase of the first parameter will finally lead to a higher level of the first parameter. An increase in the degree of GDP will increase the environmental investment, leading to higher investment in air treatment and higher air quality. Ultimately, this reduces the cost of pollution control in coal mining area, leading to a higher mining revenue and GDP. Loops (6) is a negative feedback loop. A negative feedback loop means that an increase of the first parameter will finally lead to a lower level of the first parameter. An increase in the degree of environmental investment will increase the investment in air pollution, leading to higher air quality and ecological environment quality in coal mining areas. Ultimately, this leading to a lower environmental investment.

### 2.2. Flow Diagram of Ecological Environment Subsystem in coal Mining Area

**Assumption**: To illustrate the results more clearly, we assume that five subsystems are interdependent and independent of each other. As we all know, in each subsystem, the environmental quality will be influenced by other subsystems in the real world. Here, to simplify the model, we assume that the environmental quality is determined only by this subsystem, not influenced by other subsystems.

Based on the analysis of the causal relationship of ecological environment in coal mining area, we can establish five subsystems as follows:

#### 2.2.1. Atmospheric Subsystem

**Figure 2:**
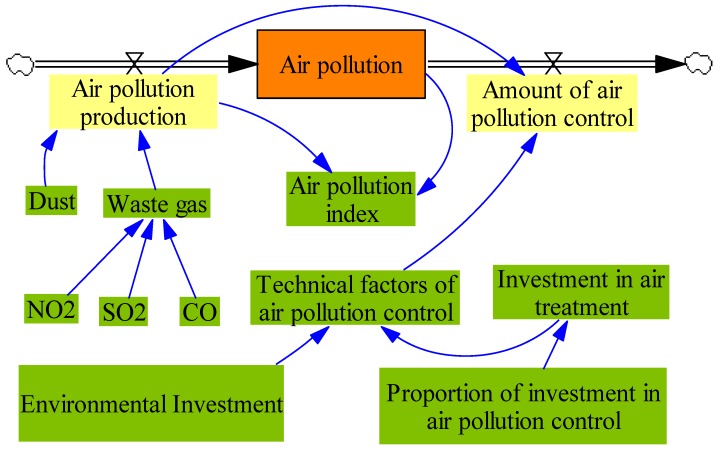
The system flow diagram of atmospheric subsystem.

The atmospheric subsystem is an important part of the ecological environment system of coal mining areas, which plays an important role in improving the air quality of coal mining areas. The system flow diagram is presented in Figure 2.

In Figure 2, air pollution is the state variable, air pollution production and the amount of air pollution control are the rate of the variables. Air pollution means the area of the air pollution. It is equal to air pollution production minus the amount of air pollution control. As can be seen from Figure 2, air pollution production is consisted of dust and waste gas. The waste gas is consisted of $NO_{2}$, $SO_{2}$ and CO. In this model, to simplify the analysis, according to the work [27,28,29,30], the equations in this model are shown in Equations (1)–(6). As shown in Equations (2)–(4), the amount of air pollution production is equal to the amount of dust plus the amount of waste gas, and the amount of the waste gas is equal to the amount of $NO_{2}$, $SO_{2}$ and CO. The amount of air pollution control is meant the amount of air pollution that is treated, the value of the amount of air pollution is equal to (Air pollution producation).K×(Technical factors of air pollution control). Technical factors of air pollution control is depended on the investment in air pollution, the higher the value of the investment in air pollution, the higher the value of technical factors of air pollution control. Equations about the Figure 2 are presented as follows:

(1) $$\begin{array}{ll} L:\;(Air\;pollution).K= & (Air\;pollution\;production).J+DT\times ((Air\;pollution\;production).JK\;-\\ & (Amount\;of\;air\;pollution\;control).JK). \end{array}$$

(2) $$\begin{array}{l} R:\;(Air\;pollution\;production).JK=(Dust).J+(Waste\;gas).J. \end{array}$$

(3) $$\begin{array}{ll} R:\;(A\mathrm{mount}\;of\;air\;pollution\;control).\;JK= & (Air\;pollution).K\times (Technical\;factors\;of\;air\\ & pollution\;control) \end{array}$$

(4) $$Waste\;gas=NO_{2}+SO_{2}+CO$$

(5) $$\begin{array}{ll} Technical\;factors\;of\;air\;pollution\;control= & Investment\;in\;air\;treatment/Environmental\\ & investment \end{array}$$

(6) $$\begin{array}{ll} Investment\;in\;air\;treatment= & Proportion\;of\;investment\;in\;air\;pollution\;control\times \\ & Environmental\;investment \end{array}$$

In Equations (1)–(3), L is the equation of state, R is the equation of rate. DT is the time interval. (Air pollution).K is the amount of air pollution in the current period. (Air pollution production).J is the amount of air pollution production at the moment J. (Air pollution production).JK and (Amount of air pollution control).JK respectively represent the amount of air pollution production and air pollution control in the period JK.

#### 2.2.2. Solid Waste Subsystem

**Figure 3:**
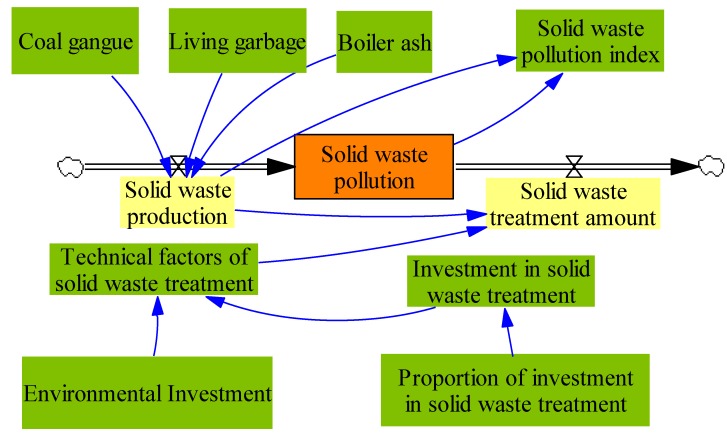
The system flow diagram of solid waste subsystem.

Solid waste subsystem is also very important for ecological environmental quality in coal mining areas. The system flow diagram is presented in Figure 3.

In Figure 3, solid waste pollution is the state variable, solid waste production and solid waste treatment amount are the rate of the variables. Here, solid waste treatment amount means the amount of solid waste that is treated. Similar to the analysis in Figure 2, here, according to the work [27,28,29,30], the equations about the Figure 3 are presented as follows:

(7) $$\begin{array}{ll} L:\;(Solid\;waste\;pollution).K= & (Solid\;waste\;production).J+DT\times ((Solid\;waste\;\\ & production).JK\;-\;(Solid\;waste\;treatment\;amount).JK). \end{array}$$

(8) R: (Solid waste production).JK=(Coal gangue). J+(Living garbage). J+(Boiler ash).J.

(9) $$\begin{array}{ll} R:\;(Solid\;waste\;treatment\;amount).\;JK= & (Solid\;waste\;production).K\times (Technical\;factors\;of\\ & Solid\;waste\;treatment) \end{array}$$

(10) $$\begin{array}{ll} Technical\;factors\;of\;solid\;waste\;treatment= & Investment\;in\;solid\;waste\;treatment/\\ & Environmental\;investment \end{array}$$

(11) $$\begin{array}{ll} Investment\;in\;solid\;waste\;treatment= & Proportion\;of\;investment\;in\;solid\;waste\;treatment\times \\ & Environmental\;investment \end{array}$$

#### 2.2.3. Water Resource Subsystem

**Figure 4:**
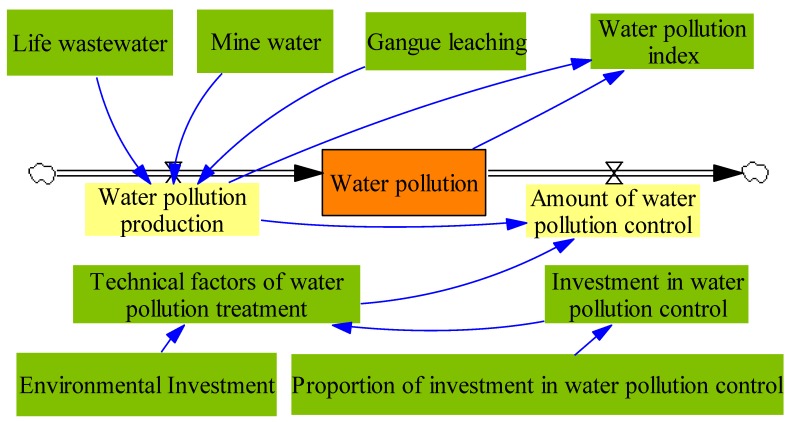
The system flow diagram of water resource subsystem.

Water resource subsystem is established in this section. The system flow diagram is presented in Figure 4.

In Figure 4, water pollution is the state variable, water pollution production and the amount of water pollution control are the rate of the variables. Here, amount of water pollution control means the amount of water pollution that is treated. According to the literature [27,28,29,30], the main equations concerning Figure 4 are presented as follows:

(12) $$\begin{array}{ll} L:\;(Water\;pollution).K=(Water & pollution\;production).J+DT\times ((Water\;pollution\\ & production).JK\;-\;(Amount\;of\;water\;plooution\;control).JK). \end{array}$$

(13) $$\begin{array}{ll} R:\;(Water\;pollution\;production).JK= & (Coal\;gangue).J+(Life\;wastewater).J+\\ & (Mine\;water).J\;+\;(Gangue\;leaching).J. \end{array}$$

(14) $$\begin{array}{ll} R:\;(Amount\;of\;water\;pollution\;control).JK= & (Water\;pollution\;production).K\times \;(Technical\\ & factors\;of\;water\;pollution\;treatment) \end{array}$$

(15) $$\begin{array}{ll} Technical\;factors\;of\;water\;pollution\;treatment= & Investment\;in\;water\;pollution\;control/\\ & Environmental\;investment \end{array}$$

(16) $$\begin{array}{ll} Investment\;in\;water\;pollution\;control= & Proportion\;of\;investment\;in\;water\;pollution\;control\times \\ & Environmental\;investment \end{array}$$

#### 2.2.4. Vegetation Cover Subsystem and Land Reclamation Subsystem

**Figure 5:**
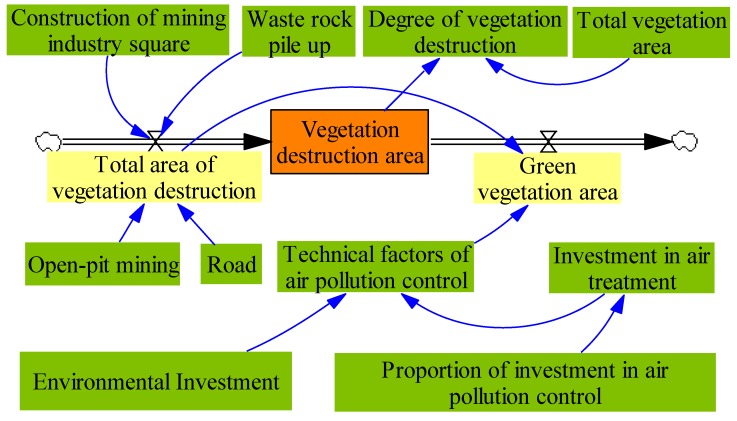
The system flow diagram of water resource subsystem.

**Figure 6:**
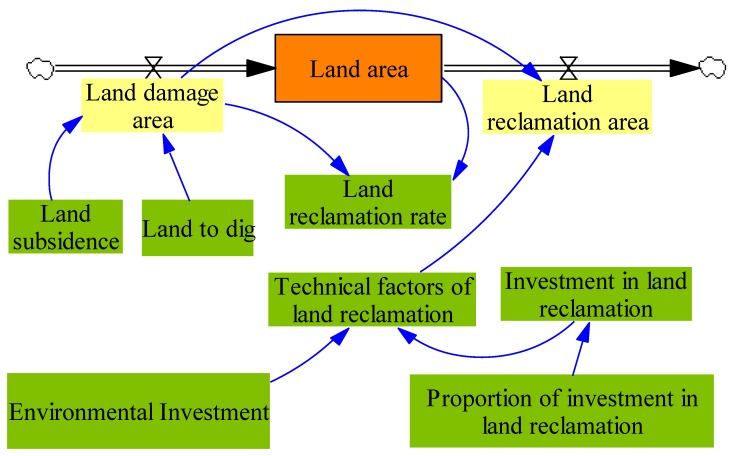
The system flow diagram of water resource subsystem.

To simplify, similar to the above analyses, we can get the vegetation cover subsystem and land reclamation subsystem. The system flow diagram of vegetation cover subsystem is presented in Figure 5, then, the system flow diagram of land reclamation subsystem is presented in Figure 6.

### 2.3. General System Flow Diagram of Ecological Environment in Coal Mining Area

**Figure 7:**
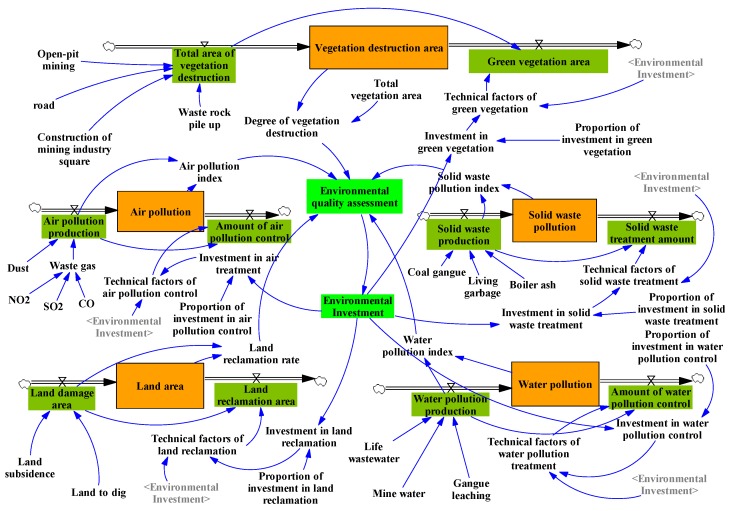
The system flow diagram of the general system.

Based on the above analyses, the general system flow diagram of ecological environment in coal mining area is established in Figure 7. As can be seen from Figure 7, the five subsystems are combined with the variable “Environmental investment”. Here, to simplify the analysis, as can be seen from Equation (A11), to find a better allocation that can get the best ecological environmental quality in coal mining areas, we use the variable “Environmental quality assessment” to show the ecological environmental quality in coal mining areas. We assume that the value of it is the average quality of the five sub-systems. The higher the value of environmental quality assessment, the higher the environmental quality in coal mining areas. All Equations left in the general system are shown in Appendix A.

## 3. A case Study in China

In the model, we set the initial value as INITIAL-TIME = 0, FINAL-TIME = 24, TIME-STEP = 1. Units for Time: Month. Integration Type: Euler. Then, a case study in China is presented.

### 3.1. Data

In this section, a case study of coal mining area in Shanxi Province is presented. The data is referenced by Ministry of Natural Resources of the People’s Republic of China and Shanxi provincial environmental protection department. The parameters of this model include state variable, rate of the variable, instrumental variable and the constant. Then, the specific initial variables are presented in Table 1.

### 3.2. Simulations

In this section, we select five indexes, namely, proportion of investment in green vegetation, proportion of investment in air pollution control, proportion of investment in land reclamation, proportion of investment in water pollution control and proportion of investment in solid waste treatment, as the control parameters for simulation control. Then, the following five regulatory schemes are presented in Table 2.

**Figure 8:**
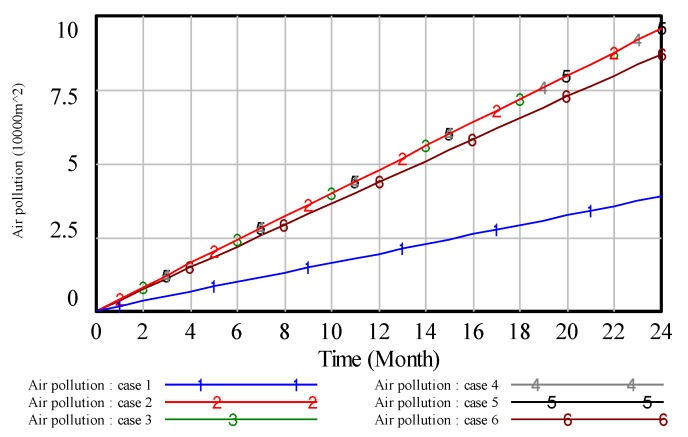
The simulation result of air pollution control.

**Figure 9:**
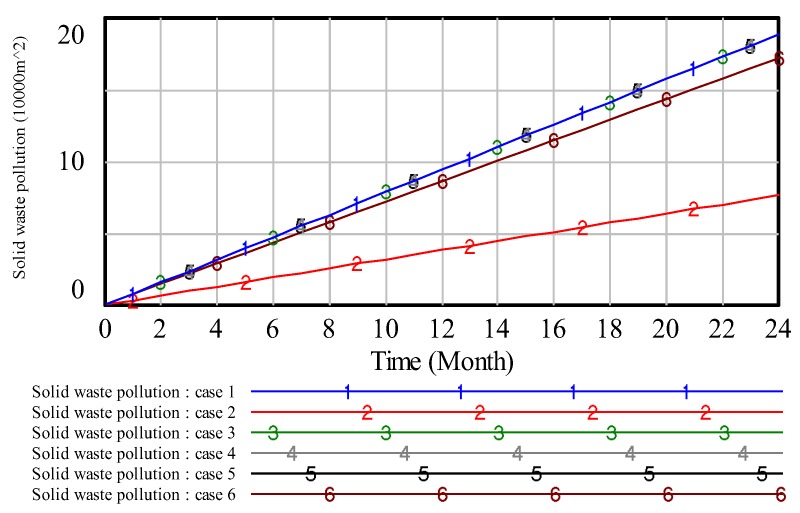
The simulation result of solid waste pollution.

**Figure 10:**
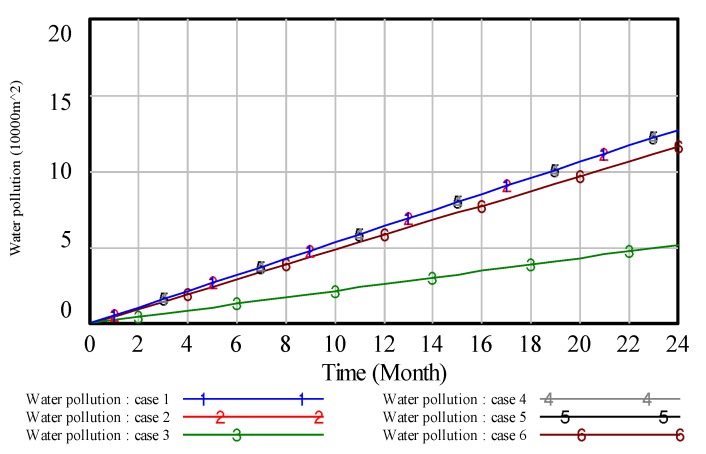
The simulation result of water pollution.

**Figure 11:**
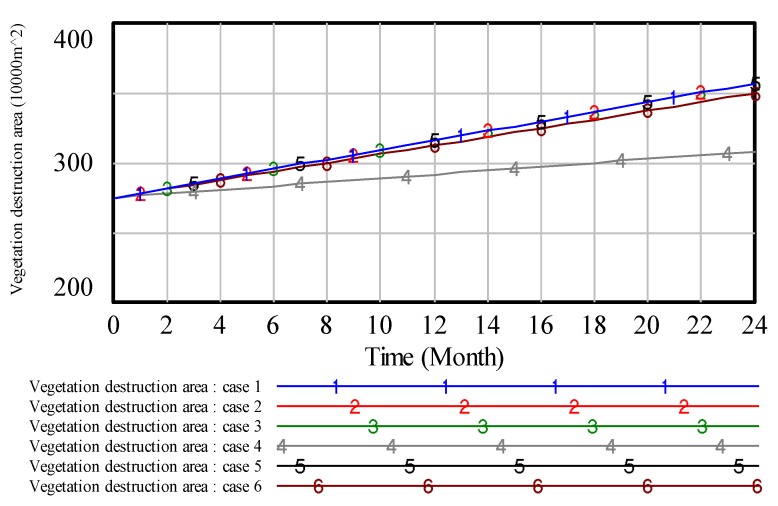
The simulation result of total vegetation destruction area.

**Figure 12:**
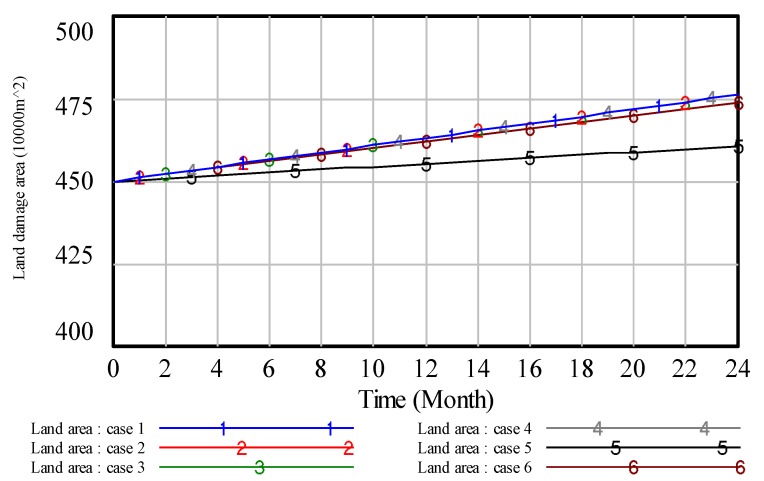
The simulation result of land damage area.

**Figure 13:**
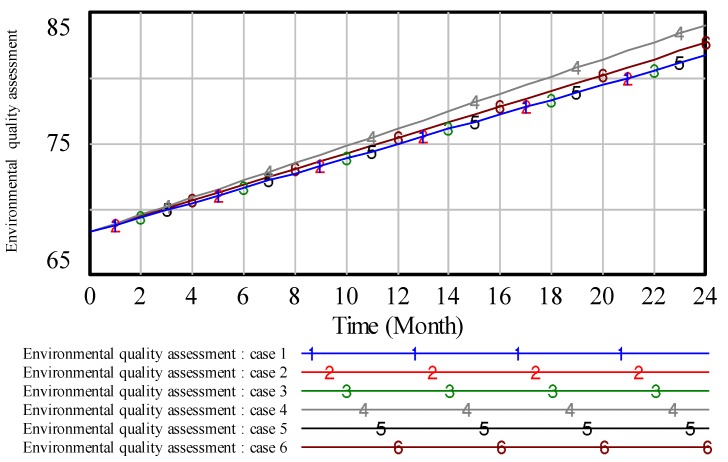
The simulation result of environmental quality assessment.

To illustrate the results, we analyze the environmental pollution control of coal mining areas from six aspects: Total vegetation destruction area, solid waste pollution, water pollution, land area, air pollution and environmental quality assessment. Then, the simulation results are presented in Figure 8, Figure 9, Figure 10, Figure 11, Figure 12 and Figure 13.

## 4. Results

### 4.1. Sensitivity Analysis

In Figure 8, when the value of ‘proportion of investment in air pollution control’ is 0.4, curve 1 shows the lowest value compared with other curves, which indicates that high investment in air pollution control will get a high effect in air pollution control. Similar to the analysis of Figure 8, we can obtain the same result on other pollution control. However, compared with the above figures, these results reveal that the effect of the high proportion of investment in green vegetation is obvious.

### 4.2. Results Analysis

From the above analyses, we can see that the five subsystems are combined with the variable “Environmental investment”. Therefore, the proportion of environmental investment in each subsystem under different cases will determine the overall environmental quality.

In case 1, we assume that the proportion of investment in air pollution control is higher than the other cases, the result is shown in Figure 8. From Figure 8, we can see that on the same time line, the lower the value of air pollution, the better effect in dealing with the problem of air pollution. Thus, we can see that case 1 is the best allocation in dealing with the problem of air pollution. That is to say, if we put a higher proportion of investment in air pollution, we can get a better effect in dealing with the problem of air pollution. In case 2, we know that the proportion of investment in solid waste treatment is higher than the other cases, the result is shown in Figure 9. From Figure 9, we can obtain that on the same timeline, the lower the value of solid waste pollution, the better effect in handling the problem of solid waste pollution. Therefore, we can see that case 2 is the best allocation in dealing with the problem of solid waste pollution. In other words, if we put a higher proportion of investment in solid waste pollution, we can get a better effect in dealing with the problem of solid waste pollution.

Similar to this method of the above analyses, we can also get the following conclusions. In case 3, the result is shown in Figure 10. The results show that, if we put a higher proportion of investment in water pollution, we can get a better effect in dealing with the problem of water pollution control. In case 4, the result is shown in Figure 11. The lower the value of vegetation destruction area comes with the better effect in handling the problem of vegetation destruction. In case 5, the result is shown in Figure 12. From Figure 12, we can see that on the same timeline, the lower the value of land damage area, the better effect in dealing with the problem of land damage. Thus, if we put a higher proportion of investment in land reclamation, we can get a better effect in solving the problem of land damage. In case 6, we know that the proportion of investment in every subsystem is equal. However, it can’t get the best environmental quality in coal mining areas.

As can be seen from Figure 13, on the same timeline, the higher the value of environmental quality assessment, the higher the environmental quality in coal mining areas. As such, case 4 can get the highest environmental quality. Case 6 have the second environmental quality. Case 1, 2, 3, 5 will get the lowest environmental quality compared with the other two cases.

Combined with the above figures, some conclusions are drawn as follows: If we want to get a better effect in dealing with some single pollution, we often need to input a huge increase in the proportion of investment in some subsystems. From all the six cases, case 4 is the optimal solution in the whole environmental quality. That is, the best improvement of mining environment can be achieved by distributing the treatment cost highly on the proportion of investment in green vegetation. Therefore, to get a better environmental quality in coal mining areas, we should focus on strengthening the restoration of vegetation damage. As such, combining the common sense, we can say that green vegetation is a sample and straight way to improve the environmental quality.

## 5. Discussion

In this paper, we presented a SD model for ecological environmental management in coal mining areas. we discussed the problem on “Given the stable investment for ecological environmental management in coal mining areas, how to find a better allocation that can get the best ecological environment quality in coal mining areas”. The result shows that, to get a better environmental quality in coal mining areas, we should focus on strengthening the restoration of vegetation damage.

Compared with other related studies, the findings of this paper have some important contributions among the perspective of the research, methods and results. These specific contributions of this research are shown as follows:

Firstly, in terms of the perspective of the research, the development of the environmental quality in coal mining areas has received much attention of a lot of scholars and governments [1,2,3,4,5,6,7]. The objective of these studies mainly introduced the development of the environmental quality or the existed environmental problems in coal mining areas. They also introduced some policies on how to treat these environmental problems. However, they didn’t give some measures on how to solve these problems can get the more effective results. For example, Zhengfu et al. [2] introduced several environmental problems of subsystems in the coal mining areas, they just listed these problems without any critical advices on how to solve these problems can get the best effect in the coal mining areas. Obviously, these studies can be more meaningful if the scholars can solve some problems in detail, instead of talking about protecting the environment in general. Therefore, in this study, our objective is to solve a specific problem on how to find a better allocation that can get the best ecological environment quality in coal mining areas when the stable investment for ecological environmental management in coal mining areas is given. This is a more meaningful and more special problem to be solved.

Secondly, in terms of methods, SD is a method of modeling to ignore the details of a system and produce a general representation of a complex system. It has been widely used in strategic and policy-making modeling and simulation, such as such as environment, supply chains, water management, transportation and so on [20,21,22,23,24,25,26,27,28,29]. As Zhengfu et al. [2] proposed, the coal mining areas is a general system combined with many subsystems. Therefore, according to our research problem, it is very appropriate to build a general model with the method of SD. This study is our first try to conduct a SD model to deal with this problem. Combined with the results of other related studies, the findings in this paper have more theoretical significance and practical value in reality.

Thirdly, in terms of results, other scholars mainly based on the qualitative and theoretical analysis, lacking effective methods and data support. This study is based on the SD model, then, we present a real case in China to make the result more convincing.

There are also some weaknesses in this paper. Some limitations are listed as follows:

The simulation results are based on predefined scenarios in this paper. We don’t consider all the possible scenarios. For the convenience of comparisons, we simply choose six cases. We will refine all cases and examine their effects on this problem in the future work. The system dynamics model is built based on assumptions and simplifications of the real systems. It can be applied to ecological environmental management in coal mining areas for many different countries. However, the conclusions are drawn from the simulations only in six cases. Therefore, they have not been validated to the best case. We plan to validate our models with more cases and find the best case to deal with ecological environmental management in coal mining areas. This will make the model more promising.

Moreover, in the future work, we can simplify consider the following cases: We can control two or more variables unchanged, and study the change of the overall environmental quality when the variables left increase or decrease, this is a more complicated situation.

## 6. Conclusions

This paper presents a SD model for ecological environmental management in coal mining areas. First, we regard the coal mining areas as a whole system. Then, we divide it into five subsystems, namely: Atmospheric subsystem, solid waste subsystem, water resource subsystem, vegetation cover subsystem and land reclamation subsystem. Given the stable investment for ecological environmental management in coal mining areas, our objective is to find a better allocation that can get the best ecological environment in coal mining areas. Then, we present six allocations of the investment for ecological environmental management in coal mining areas. The results show that, in allocation 1, we can get the best ecological environment in coal mining areas. That is, the best improvement of mining environment can be achieved by distributing the treatment cost highly on the proportion of investment in green vegetation.

From the above results, some recommendations are presented to the policy of the government. Each subsystem is very important to the environmental quality of the coal mining area. However, to get a better environmental quality in coal mining areas, they should focus on strengthening the restoration of vegetation damage. The government should take measures to handle this problem, besides, they cannot ignore the importance of other subsystems to the environment. Moreover, enterprises should shoulder the important responsibility of protecting the environment in coal mining during the process of production.

This paper studies a SD model for ecological environmental management in coal mining areas under five cases. However, the real situation in the real world is more complicated. So the further research we can present more cases to examine the results. Moreover, in the future work, we should also focus on simultaneous changes in all the variables to determine the global sensitivity of the model.

## Figures and Tables

**Table 1:** The main parameters in the model.

Variable Types	The Variable Name	The Initial Value	Unit
Constant	Construction of mining industry square	30,000	m^2^
	Waste rock pile up	9833	m^2^
	Open-pit mining	1500	m^2^
	Road	80	m^2^
	Environmental Investment	50	Ten thousand yuan
	Total vegetation area	2645.2	10,000 m^2^
	Dust	3310	m^2^
	Waste gas	1514	m^2^
	Land subsidence	4400	m^2^
	Land to dig	8800	m^2^
	Coal gangue	8750	m^2^
	Boiler ash	298	m^2^
	Life waste water	980	m^2^
	Mine water	5392	m^2^
	Gangue leaching	73	m^2^
State variables	Vegetation destruction area	275	10,000 m^2^
	Air pollution	300	m^2^
	Land area	450	10,000 m^2^
	Solid waste pollution	25.6	m^2^
	Water pollution	35	m^2^

**Table 2:** The five regulatory schemes.

Regulatory Schemes	Case 1	Case 2	Case 3	Case 4	Case 5	Case 6
Proportion of investment in air pollution control	0.4	0.15	0.15	0.15	0.15	0.2
Proportion of investment in solid waste treatment	0.15	0.4	0.15	0.15	0.15	0.2
Proportion of investment in water pollution control	0.15	0.15	0.4	0.15	0.15	0.2
Proportion of investment in green vegetation	0.15	0.15	0.15	0.4	0.15	0.2
Proportion of investment in land reclamation	0.15	0.15	0.15	0.15	0.4	0.2

## Appendix A. All Equations Lest in the General System

(A1) $$\begin{array}{l} L:\;(Vegetation\;destruction\;area).K=(Total\;area\;of\;vegetation\;destruction\;area).J+\\ DT\times ((Total\;area\;of\;vegetation\;destruction\;area).JK\;-\;(Green\;vegetation\;area).JK). \end{array}$$

(A2) $$\begin{array}{r} R:\;(Total\;area\;of\;vegetation\;destruction).JK=(Open-pit\;\min ing).J\;+(Road).\;J+\\ (Construction\;of\;\min ing\;industry\;square).J+(Waste\;rock\;pile\;up).J. \end{array}$$

(A3) $$\begin{array}{c} R:\;(Green\;vegetation\;area).JK=(Total\;area\;of\;vegetation\;destruction).K\times (Technical\\ factors\;of\;air\;pollution\;control) \end{array}$$

(A4) $$\begin{array}{c} Technical\;factors\;of\;green\;vegetation=Investment\;in\;green\;vegetation/Environmental\\ investment \end{array}$$

(A5) $$\begin{array}{c} Investment\;in\;green\;vegetation=Proportion\;of\;investment\;in\;green\;vegetation\times \\ Environmental\;investment \end{array}$$

(A6) $$\begin{array}{c} L:\;(Land\;area).K=(Land\;damage\;area).J+DT\times ((Land\;damage\;area).JK\;-\\ (Land\;reclamation\;area).JK). \end{array}$$

(A7) $$\begin{array}{ll} R:\;(Land\;damage\;area).JK & =(Land\;subsidence).J\;+(Land\;to\;dig).\;J. \end{array}$$

(A8) $$\begin{array}{ll} R:\;(Land\;reclamation\;area).\;JK= & (Land\;damage\;area).K\times (Technical\\ & factors\;of\;land\;reclamation) \end{array}$$

(A9) $$\begin{array}{c} Technical\;factors\;of\;land\;reclamation=Investment\;in\;land\;reclamation/Environmental\\ investment \end{array}$$

(A10) $$\begin{array}{c} Investment\;in\;land\;reclamation=Proportion\;of\;investment\;in\;land\;reclamation\times \\ Environmental\;investment \end{array}$$

(A11) $$\begin{array}{r} Environmental\;quality\;assessment=(Solid\;waste\;pollution\;index+Land\;reclamation\;rate\\ +Air\;pollution\;index+Degree\;of\;vegetation\;destruction+Water\;pollution\;index)/5 \end{array}$$
